# Custom-Made Articulating Spacer (CUMARS): The Resolution of Periosteal Reaction and Femur Remodelling in Periprosthetic Hip Infection

**DOI:** 10.7759/cureus.41669

**Published:** 2023-07-10

**Authors:** Rosalind Wong, Azlina A Abbas, Khairul A Ayob, Haidar Nasuruddin, Veenesh Selvaratnam

**Affiliations:** 1 Joint Reconstruction Unit (JRU) National Orthopedic Centre of Excellence for Research and Learning (NOCERAL) Department of Orthopedic Surgery, Faculty of Medicine, Universiti Malaya, Kuala Lumpur, MYS

**Keywords:** custom-made articulating spacer, revision arthroplasty, hip reconstruction, cumars, tha, total hip arthroplasty, pji, periprosthetic joint infection

## Abstract

Periprosthetic joint infection (PJI) is one of the most common complications after total hip arthroplasty (THA). Two-stage revision surgery is one of the treatment options for PJI, however, it has been associated with poor patient tolerance, reduced patient mobility, and periarticular tissue contracture leading to difficulty during second-stage reconstruction. The custom-made articulating spacer (CUMARS) was developed to provide an alternative that is better tolerated and to reduce the complexity of second-stage reconstruction. This study details the treatment of a patient with PJI post-THA with significant periosteal reaction using a CUMARS construct, which enabled immediate post-operative weight bearing, eventual eradication of infection, restoration of femoral bone stock, and avoidance of second-stage reconstruction.

## Introduction

Periprosthetic joint infection (PJI) post-total hip arthroplasty (THA) is a serious burden for healthcare systems, treating surgeons, and affected patients. Management is still controversial and various treatment options are available, one of which is a two-stage revision surgery [1]. The conventional method was poorly tolerated by patients due to multiple short-term complications and reconstruction of the joint was difficult at the second stage. The custom-made articulating spacer (CUMARS) construct was developed in an effort to reduce short-term complications and aid second-stage reconstruction [2]. We report a case of PJI post-THA with significant periosteal reaction which was treated with the CUMARS construct and the outcome two years post-CUMARS construct. We also wish to highlight the remarkable resolution of periosteal reaction and remodeling of the femur.

## Case presentation

A 61-year-old lady, who underwent left THA with a hybrid hip replacement (uncemented acetabular component and a cemented stem) one year prior in a different center, presented to us with inability to walk due to left hip pain, which was progressively worsening over the course of five months. She was unable to bear weight on her left lower limb but had no systemic signs or symptoms of infection.

**Figure 1:**
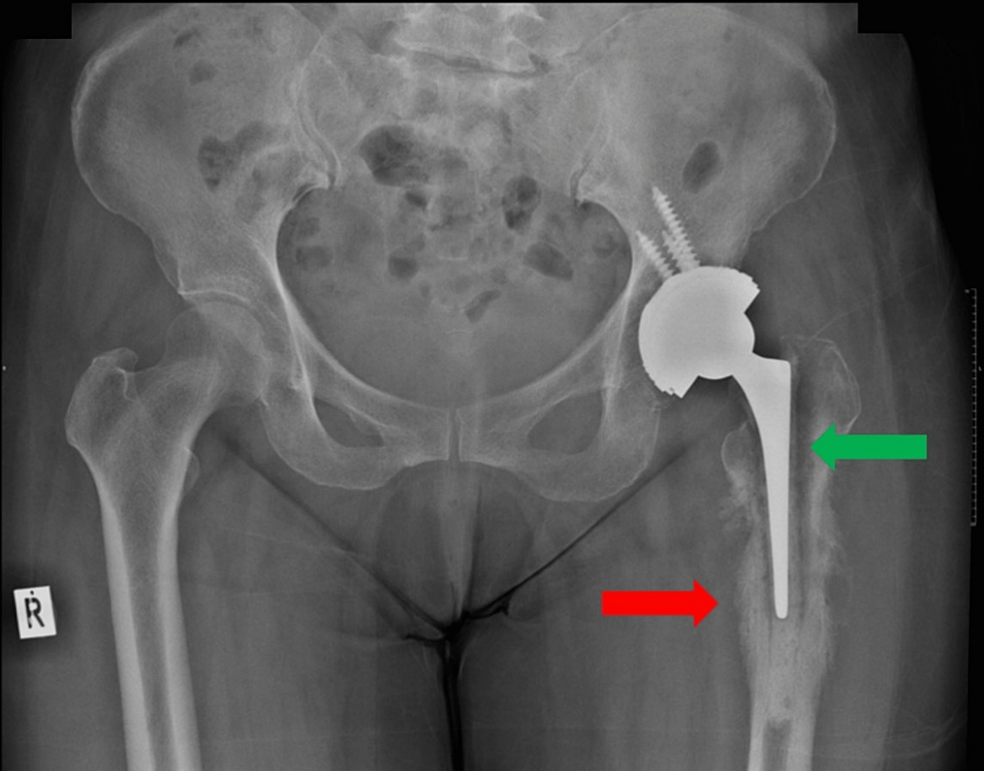
Pelvis AP radiograph showing periosteal reaction at the left proximal femur (red arrow) and loosening of the femoral component (green arrow). AP: anteroposterior

Plain radiographs of her pelvis and left hip showed sunburst appearance of the proximal left femur with loosening of the femoral stem (Figure 1). Her inflammatory markers were raised and readings were as follows: C-reactive protein (CRP) was 48.2 mg/L and erythrocyte sedimentation rate (ESR) was 88 mm/h. Contrast-enhanced computed tomography (CT) scan of the pelvis and bilateral femurs showed no collection. Aspiration of her left hip was done but unfortunately did not grow any organism.

She underwent a revision left THA using the CUMARS construct. Intra-operatively, there was inflamed tissue surrounding her left hip joint and her left proximal femur was thickened due to the periosteal reaction. There was also a sinus at the anterior cortex of her proximal femur with slough tissue in the acetabulum and the femoral medullary cavity as well as minimal pus within the femoral canal. Pus and tissue samples were taken and sent for cultures. A limited extended trochanteric osteotomy (ETO) was done to remove all cement in the femoral canal. Cerclage wiring was utilized to fix the ETO.

Four grams of Vancomycin and 4 g of ceftazidime were mixed into each pack of 40 g bone cement to ensure broad spectrum coverage of Gram-positive and Gram-negative organisms as we could not identify an organism pre-operatively. Three cement packs were used in total for the implantation, one cement pack for the acetabular component and two for the femoral component. On the acetabular side, a Rimfit X3 (Kalamazoo, MI: Stryker Orthopedics) was cemented while on the femoral side an Exeter V40 (Kalamazoo, MI: Stryker Orthopedics) long 205 mm stem was cemented.

Post-operatively, she was given intravenous (IV) Unasyn 3 g TDS and IV vancomycin 1 g BD empirically as per our microbiology protocol for revisions for infections until definitive cultures were available. All intra-operative samples grew *Streptococcus agalactiae*, which was sensitive to penicillin and ampicillin. Her antibiotics were then switched to IV C-penicillin and oral rifampicin. She was switched to oral ampicillin and oral rifampicin for a total of 12 months after completing two weeks of IV antibiotics. Prior to her discharge two weeks post-surgery, she was able to ambulate pain-free using a walking frame.

**Figure 2:**
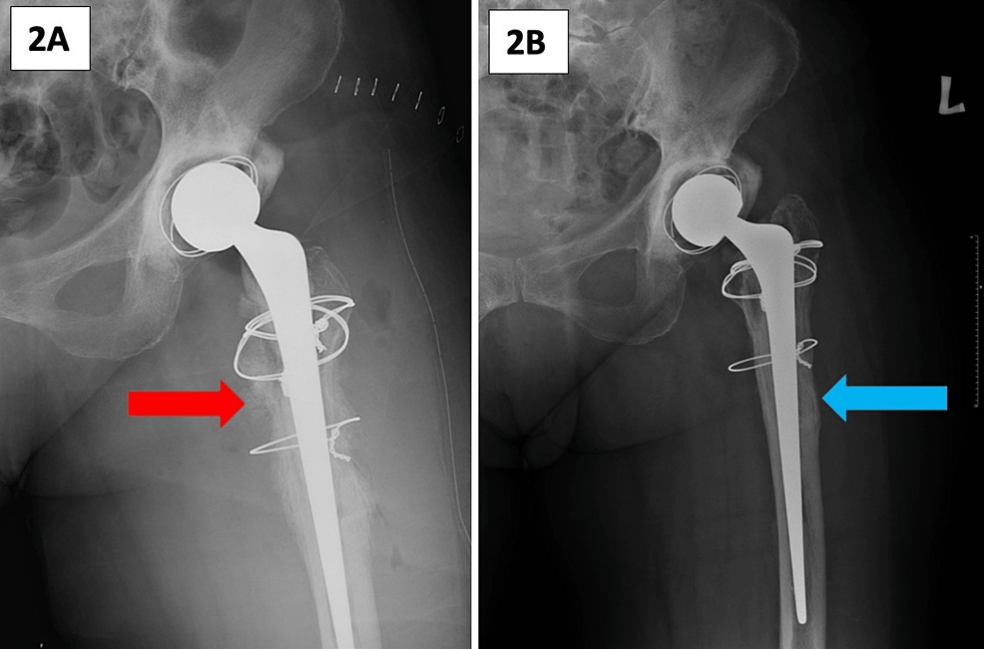
Left hip radiograph immediately post-surgery showing that the periosteal reaction (red arrow) is still present (A); left hip radiograph at one year post-surgery showing remodeling of the proximal femur (blue arrow) (B).

Serial radiographs obtained during clinic visits showed that the previously seen periosteal reaction (Figure 2A) had resolved completely and the left proximal femur had remodeled as seen on the radiograph repeated at one-year post-surgery and after completion of antibiotics (Figure 2B). She had been well throughout all her clinic visits with no complaints of pain and was able to walk independently without any aids. She is currently infection-free one year after the completion of antibiotics.

## Discussion

PJI is one of the most devastating complications following THA and remains a significant challenge for arthroplasty surgeons as well as a cause of distress for affected patients. Despite extensive studies on how best to manage PJI, it is still controversial and one of the options for treatment is a two-stage revision surgery. It involves removal of the implants, debridement of all nonviable soft tissue, and insertion of an antibiotic-loaded cement spacer in the first stage, and subsequently reimplantation of a hip prosthesis in the second stage after the infection has settled [1].

The CUMARS construct popularized by the Exeter Hip Unit in the United Kingdom initially involved a loosely cemented slim femoral stem and a large polyethylene cup with antibiotic-loaded cement [2]. This proved to be advantageous over a conventional excision arthroplasty and non-articulating cement spacers as it allowed the patient early joint mobilization and acceptable amounts of weight bearing between stages of revision, reduced periarticular soft tissue contracture while still delivering high-dose of antibiotics locally, greatly simplifying the second stage procedure [2]. Tsung et al. later found that a stable CUMARS construct could provide pain-free mobilization if the infection was eradicated, allowing indefinite delay and potentially eliminating the need for the second-stage procedure [3]. The method of implantation was then modified to create a long-term spacer - a construct that had a more well-fixed femoral stem and was used in patients whose implants were intended to remain in place indefinitely. All patients in whom the long-term spacers were used, retained their CUMARS construct without proceeding to the second stage revision within the two-year period of follow-up, potentially reducing the cost of resources and the patients’ morbidity associated with repeat surgery [3]. In our center, we are using well-fixed CUMARS to treat THA PJI as we get more confident with this technique.

In this case, we performed an ETO to remove all the cement as the bone cement interface was loose. In the presence of infection where the femoral bone cement interface is intact, a cement-in-cement revision with antibiotic-loaded cement can be done on the femoral side [4].

The usage of antibiotic-loaded bone cement is also a significant component in the treatment of PJI post-THA. However, elution of antibiotics from bone cement over time will result in increased porosity, essentially reducing the mechanical properties of the bone cement, which is a factor to consider in long-term implant fixations as in the case of retained CUMARS constructs. In a study conducted by Wu et al., it was found that the addition of antibiotics exceeding 4.8% of a pack of 40vg bone cement, reduced the compression strength to 66-69 MPa [5], which is lower than the minimum compressive strength of set and cured bone cement of 70MPa as set by the ISO 5833:2002 [6]. At present, the effect of antibiotic-loaded bone cement on long-term fixation is not yet known and requires further study as it would affect the longevity of the CUMARS construct. According to a study by Springer et al. in 2004, up to 4 g of vancomycin and 4.8 g of gentamicin can be added to each pack of 40 g bone cement without any clinical adverse effects for the treatment of PJI [7]. It has also been reported that the highest ratio of antibiotics per 40 g of bone cement that can be introduced is 8 g, and this will still allow the cement to be molded and formed [8]. Despite some reports of acute kidney injury (AKI) after insertion of high-dose antibiotic cement spacers [9,10], it has been reported to be safe and the risk of AKI was not due to the antibiotics impregnated into the spacer [11].

The type of antibiotics placed in cement spacers for treatment of PJI post-THA should be tailored to the sensitivity of the infective organism. Aspiration of the affected joint and pre-operative blood cultures is helpful in determining the choice of antibiotics to be used for the cement spacer to target the causative organism and ensure higher possibility of eradication of infection [12]. Unfortunately, pre-operative hip aspiration culture of our patients did not yield an organism. Therefore, we added vancomycin and ceftazidime to the cement mix. A study by Hsu et al. in 2017 showed that bone cement mixed with high doses of vancomycin and ceftazidime exhibited antibacterial activity against methicillin-sensitive *Staphylococcus aureus* (MSSA), methicillin-resistant *Staphylococcus aureus* (MRSA), *Staphylococcus epidermidis*, *Pseudomonas aeruginosa, *and *Escherichia coli *for as long as or longer than bone cement mixed with other antibiotic combinations, provided fast and abundant elution of the antibiotics [13]. Our patient was successfully treated with the antibiotic combination of vancomycin and ceftazidime mixed in bone cement. She has been able to avoid a second revision surgery by retaining her CUMARS.

The application of Wolff’s law, coupled with the biomechanics of the collarless, tapered, and polished femoral stem which undergoes controlled subsidence, was observed in our patient [14]. The initial osteopenic proximal femur was able to undergo remodeling and strengthening due to the taper-slip phenomenon. Wolff’s law states that bone density changes in response to changes in the functional forces on the bone, and this could be seen in the complete remodeling of the proximal femur with progressive weight bearing after surgery. Load applied to the prosthetic joint during daily activities is supported by the production of strain due to subsidence of the stem within the cement mantle. This induces radial compression, hoop tension, and shear stress into the cement mantle, which transmits the forces to the surrounding bone, essentially loading the femur, especially proximally, leading to remodeling in accordance with Wolff’s law [6]. The resolution of periosteal reaction correlated with infection eradication. This patient is still under our outpatient follow-up to assess the long-term survival of her CUMARS construct with heavily loaded antibiotic cement.

## Conclusions

In general, the long-term outcomes of high-dose antibiotics in cement spacer in component fixation will require further studies, as existing literature has shown that the biomechanical properties of bone cement are weakened with the addition of antibiotics beyond a certain dosage. However, we can be sure that it minimizes the short-term potential complications and is effective in the eradication of infection. In this case, the taper-slip principle of the Exeter stem also helped with loading the femur and bone remodeling.
